# Moiré topography as a screening and diagnostic tool—A systematic review

**DOI:** 10.1371/journal.pone.0260858

**Published:** 2021-12-02

**Authors:** Marta Kinga Labecka, Magdalena Plandowska

**Affiliations:** 1 Department of Rehabilitation, Jozef Pilsudski University of Physical Education in Warsaw, Biala Podlaska, Poland; 2 Faculty of Physical Education and Health in Biala Podlaska, Jozef Pilsudski University of Physical Education in Warsaw, Biala Podlaska, Poland; Danube Private University, AUSTRIA

## Abstract

Diagnostic investigation can be carried out using non-radiological and non-contact methods. Moiré topography (MT) seems to be a viable alternative to radiographic research in evaluating the spine and/or trunk deviations. The aim of this systematic review was to analyze the current knowledge regarding the reliability and validity of Moiré topography as a screening and diagnostic tool. The systematic review was performed from 2010 until March 2021 in the PubMed, EBSCO, Web of Science, and Scopus databases, according to the eligibility criteria. This review fulfilled the following criteria according to the PICO system: population (children and adolescents), intervention (MT measurement), comparison (repeated MT measurements, MT compared to Cobb angle or scoliometer), outcome (reliability and validity of MT). Eight studies fulfilled the inclusion criteria for further analysis. All the studies were assessed to be of high quality. Included studies found that MT had high repeatability and high intraobserver and interobserver correlation, and correlation between MT parameters and radiographic Cobb angle ranged from moderate to high. The authors reported difficulty in defining the cut-off values for MT parameter (Surface Trunk Rotation—STR), and unsatisfactory sensitivity and specificity of MT examination. The studies did not reveal the advantage of MT as a screening method in the detection of idiopathic scoliosis in comparison to radiograph. Based on the evidence from eight studies, the results indicated moderate evidence for reliability and validity of Moiré topography as a screening and diagnostic tool. There is still no strong evidence for the accuracy of MT.

## Introduction

Postural deviations in children and adolescents are an important medical and social issue. Research indicates a disturbing phenomenon of the frequent appearance and progression of irregularities in the body posture of the youngest part of society [1–4]. Initially, they develop asymptomatically and their consequences can be felt over the next years of life. Examining children in terms of assessing their posture seems justified because serious irregularities can significantly change the quality of life, cause significant deformations in the osteoarticular system, pain, and disorders of the internal organs [5, 6]. Consequently, screening is the most important factor preventing deformity from progressing.

This problem’s significance and its universality suggest the need to develop objective methods for diagnosing body posture. The gold standard in identifying changes in the spine position is the radiographic examination [7–9]. However, the disadvantage of radiographs is that repeated exposure to ionizing radiation may be harmful to patients [10]. Children and adolescents are the groups most susceptible to the effects of radiation because their bodies have increased sensitivity to these effects, which can cause modification of genetic material [11, 12].

Diagnostic investigation can be carried out using non-contact and non-radiological methods to reduce radiation exposure. The basic method of school screening scoliosis is the Adams test (examination in forwarding bending position) using a scoliometer [9, 13]. Nowadays several non-radiographic and non-invasive methods have been proposed as a method of school screening for scoliosis. Such methods include Moiré topography (MT), raster stereography (Diers Formetric) [14, 15], 3D ultrasound imaging (the Scolioscan system) [16], and Infrared Thermography (IR thermography) [17]. MT was one of the first techniques and has been used as a method of clinical diagnosis in topographic analyzes since 1970 [18]. As a technique of testing body posture, it is easy, non-invasive, and suitable for use in schools and health care units [19]. MT is based on optical phenomena, thanks to which it enables the analysis of the shape of objects in three dimensions. The picture is created by alternating clear and dark stripes. The pattern formed by these fringes on the surface of the object is applied for subsequent analysis.

The latest study which presents a literature review regarding the main characteristics of the MT was published in 2010 [19]. MT is detecting early stages of scoliosis and different deformities of the spine. However, further research will improve the analysis of the topograms [19]. To the best of our knowledge, school scoliosis screening by MT causes controversy. Therefore, the analysis of the current research is important to improve the understanding of the MT for the prognosis of postural deviations in three planes (sagittal, frontal, and transverse).

The aim of this systematic review was to analyze the current knowledge regarding the reliability and validity of Moiré topography as a screening and diagnostic tool.

## Materials and methods

### Literature search

The systematic review was performed from 2010 until March 2021 in PubMed, EBSCO (Health Source—Consumer Edition, Health Source: Nursing/Academic Edition, Academic Search Ultimate, MEDLINE, SPORTDiscus with Full Text, AHFS Consumer Medication Information), Web of Science and Scopus databases.

The search strategy included keywords related to Moiré OR “Moiré topography” OR “Shadow Moiré” OR “Moiré technique” OR photogrammetry OR “photogrammetric method” OR “Moiré phenomenon” OR “projection Moiré” AND “body posture” AND children OR adolescent. Table 1 shows the search strategy for the PubMed database.

**Table 1:** Search strategy (PubMed).

Search	Search terms
#1	"body posture" [Title/Abstract] OR spine [Title/Abstract] OR "spine curvature*" [Title/Abstract] OR "column" [Title/Abstract] OR "trunk" [Title/Abstract] OR "trunk asymmetry" [Title/Abstract] OR "anterior-posterior" [Title/Abstract] OR "anteroposterior" [Title/Abstract] OR "frontal plane" [Title/Abstract] OR "sagittal plane" [Title/Abstract] OR “transvers* plane” [Title/Abstract]
#2	“scoliosis” [Mesh] OR scoliosis [Title/Abstract] OR “adolescent idiopathic scoliosis” [Title/Abstract] OR “idiopathic scoliosis” [Title/Abstract] OR “screening scoliosis” [Title/Abstract] OR “scoliosis evaluation” [Title/Abstract]
#3	# 1 OR #2
#4	Moiré [Title/Abstract] OR “Moiré topography” [Title/Abstract] OR “Shadow Moiré” [Title/Abstract] OR “Moiré technique” [Title/Abstract] OR photogrammetry [Title/Abstract] OR “photogrammetric method” [Title/Abstract] OR “Moiré phenomenon” [Title/Abstract] OR “projection Moiré” [Title/Abstract] OR “surface topography” [Title/Abstract]
#5	"Child" [Mesh]
#6	"Adolescent" [Mesh]
#7	child* [Text Word] OR "adolescen*"[Text Word] OR teen*[Text Word] OR schoolchildren [Text Word] OR "school children"[Text Word]
#8	#5 OR #6 OR #7
#9	#3 AND #4 AND #8

### Eligibility criteria

The studies included if they: (1) were original research, (2) were published in English, (3) were from the last 11 years, (4) included males and/or females aged under 21 with and without spine deformity, (5) reported reliability and/or validity of MT.

### Research selection and data extraction

The studies were independently searched by two reviewers (MKL, MP). The reviewers screened the identified papers and made decisions about inclusion according to the eligibility criteria. All articles were screened to identify relevant studies, first by title, secondly by abstract, and thirdly by full-text screening. The article was considered potentially significant and the full-text was reviewed if, after discussion by two independent reviewers, it could not be clearly excluded on the basis of the title and abstract [20]. Misunderstandings were resolved through discussions between researchers. Only the included studies were submitted for data extraction and methodological quality evaluation. The extracted data included author, year of publication, study population, participant characteristics. Duplicates were deleted with Endnote online.

Our review fulfilled the following criteria according to the PICO system (Population: children and adolescents; Intervention: MT measurement, Comparison: 1) repeated MT measurements, 2) MT compared to Cobb angle or scoliometer; Outcome: reliability and validity of MT. The reliability and validity of MT were assessed by calculating Pearson`s correlation coefficient (r), or False-Positive Rate (FPR), or Positive Predictive Value (PPV) and/or Negative Predictive Value (NPV), or the sensitivity and the specificity, or intraobserver and interobserver error, or Interclass Correlation Index. The analysis of the results was limited to a qualitative summary.

**Fig 1:**
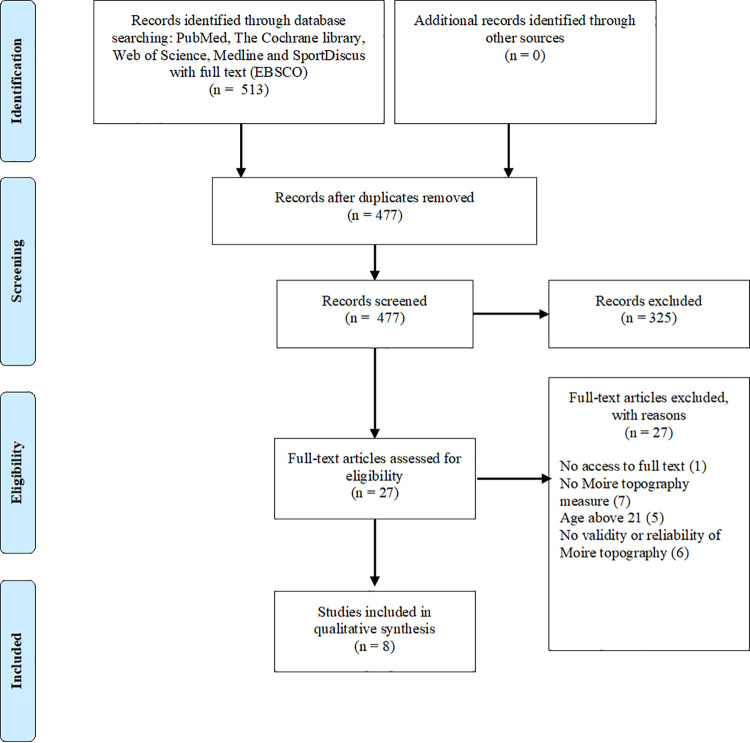
Flow chart of the included studies in this review.

Articles were removed if the study did not meet the previously specified selection criteria. The number of articles included and excluded at different phases was presented in a PRISMA flowchart (Fig 1). PRISMA guidelines were followed for this systematic review [21].

### Quality assessment

The studies were assessed for methodological quality using a 13-item critical appraisal tool developed by Brink and Louw [22], for assessing validity and reliability of results from studies (Table 2). Five items were used to assess the methodological quality of both validity and reliability studies, four items were applied for validity studies, and four items for reliability studies. Preceding the final screening, reviewers tested the methodological quality assessment of two similar articles that were not included in this review. Disagreements were resolved through discussion or consultation.

**Table 2:** Methodological quality of included studies.

Article	1	2	3	4	5	6	7	8	9	10	11	12	13	High quality ≥ 60%
Fugiel and Krynicka, 2010 [24]	y	n	y	n/a	n/a	n/a	n	n/a	y	y	y	n	y	67% (6/9)
Ueno et al., 2011 [25]	y	n	y	n/a	n/a	n/a	n	n/a	y	y	y	n	y	67% (6/9)
Chowanska et al., 2012 [26]	y	n	y	n	n	n	y	y	y	y	y	y	n	62% (8/13)
Yamamoto et al., 2015 [27]	y	n	y	n/a	n/a	n/a	n	n/a	y	y	y	n	y	67% (6/9)
Pino-Almero et al., 2016 [28]	y	y	y	n	n	n	n	n	y	y	y	y	y	62% (8/13)
Pino-Almero et al., 2017a [29]	y	n	y	n/a	n/a	n/a	n	n/a	y	y	y	y	y	78% (7/9)
Pino-Almero et al., 2017b [30]	y	n	y	n	n	y	n	n	y	y	y	y	y	62% (8/13)
Kuroki et al., 2018 [31]	y	y	y	n/a	n/a	n	y	n/a	y	n	n	n	y	67% (6/9)

1. Sample description; 2. Characteristics of the evaluators; 3. Use of gold standard for comparison (validity only); 4. Inter-evaluators blindness (reliability only); 5. Intra-evaluators blindness (reliability only); 6. Randomization of evaluators or subjects (reliability); 7. Period of time between the test collection (validity); 8. The time interval between repeated measures (reliability); 9. The studied test is not part of the gold standard (validity); 10. Description of the sampling procedures for the experimental test; 11. Description of the gold standard collection procedures (validity); 12. Description of sample loss cases; 13. Adequacy of the statistical method. y = yes; n = no; n/a = not applicable; % = final score reached by the study.

Each item was rated as yes or no or not applicable. Each study was assigned a total score, which was the sum of all positive ratings according to the methodological criteria. The reviewers considered studies to be of high quality if the methodological quality score was ≥ 60% of the maximum score, as proposed by previous studies [23].

## Results

### Search result

This literature search yielded a total of 514 articles. After removing duplicates, 477 studies remained. Based on the analysis of the titles and abstracts 27 studies were eligible for assessment by full paper. Following the full-text review of 27 articles, 8 studies [24–31] fulfilled the inclusion criteria for further analysis. The number of articles included and excluded at different phases was presented in a flowchart (Fig 1).

### Study characteristics

The total sample size of the eight included studies consisted of 1 141 645 participants, with the age range of 7–21 years. The systematic review showed studies using MT among healthy children [25–27, 30, 31] and with scoliosis [24, 28–30]. In all research enrolled as well as girls and boys. The characteristics of the included studies are described in Table 3.

**Table 3:** Characteristics of the included studies.

Author (year)	Participants	Anatomical landmarks	Parameters evaluated	Methodological procedure	Measurement of validity	Measurement of reliability	Results
Fugiel and Krynicka, 2010 [24]	58 girls with first- and second-degree scoliosis (12–14 years old)	• spinous processes from C7 to L5,	• UK parameter characterizing the location of the line spinous processes relative the vertical line from the spinous process C7	• comparison the results obtained by MT with the Cobb angle values	• Spearmen correlation		• the values of Spearman correlation indicate high or moderate correlations (0.61–0.78),
		• lower angles of the scapulas,					• high correlations were observed in thoracic and lumbar scoliosis,
		• posterior superior iliac spines					• moderate correlation was observed in thoraco-lumbar scoliosis
Ueno et al., 2011 [25]	255.875 children, boys and girls (11–14 years old)		• upper thoracic spine,	• two independent examiners assessed the asymmetry of the Moiré fringes,	• The False-Positive Rate (FPR, %)		• FPR = 35.0%,
			• thoracic spine,				• FPR in the 11- to 12-year-old group = 32.4% in girls and 59.7% in boys,
			• lumbar spine,			
			• the asymmetry of the waistline and deviation of the dorsal side of the thoracic spine (α)	• Cobb angle of the curves was measured by several very experienced spine surgeons			• FPR in the 13- to 14-year-old group = 33.0% in girls and 50.7% in boys
Chowanska et al., 2012 [26]	996 girls (9−13 years old)	• spinous processes from C7 through S1,	• Surface Trunk Rotation (STR)	• in ten girls three researchers examined the value of interobserver error for STR parameter,	• Positive Predictive Value (PPV, %),	• Intraobserver and interobserver reproducibility	• for STR parameter intraobserver error = 1.9°, interobserver error = 0.8°,
		• posterior superior iliac spines			• Negative Predictive Value (NPV, %),		• PPV was from 8.1% (STR ≥ 7°) to 48 (STR ≥ 4°) compared with scoliometer (ATR ≥ 7°),
				• in fifty girls twice by the same researcher examined the intraobserver error for the STR parameter,	• the sensitivity,		• the false positive results of MT were from 99% (STR ≥ 7°) to 98 (STR ≥ 4°) compared with scoliometer (ATR ≥ 7°),
				• comparison MT examination and scoliometer examination	• the specificity		• for STR ≥ 5° (sensitivity = 64.5% specificity = 88%),–-for STR ≥ 4° (sensitivity = 77.4%, specificity = 71.1%)
Yamamoto et al., 2015 [27]	195.149 children 11–14 years old		• Moiré fringes on each half of the back,	• position assessed by 1 nurse and 1 radiology,	• Positive Predictive Value (PPV, %),		• PPV = 33.3%,
			• shoulder height,	• MT method compared with radiograph	• The False-Positive Rate (FPR, %)		• FPR = 66.7%
			• pelvic tilt,				
			• waistline differences				
Pino-Almero et al., 2016 [28]	31 patients with idiopathic scoliosis, 7−17 years old	16 anatomical landmarks:	• Posterior Trunk Symmetry Index (POTSI),	• radiographic and topographic evaluation on two separate times (6 months to 1-year interval),	• Pearson’s linear correlation coefficient r and the coefficient of determination (R^2^)	• Intraobserver and interobserver reproducibility–Interclass Correlation Index	• the Intraclass Correlation Index for the intraobserver and interobserver correlation was perfect (within limits 0.959–0.987 for measures parameters),
		• corners of the shoulders,	• Horizontal Plane Deformity Index (DHOPI),	• these measurements were repeated by the same researcher and second observer (specialist physician in orthopedic surgery)			• revealed significant correlation (p < 0.01) between the Cobb angle with DHOPI (r = 0.769; R^2^ = 0.591) and POTSI (r = 0.539; R^2^ = 0.291),—agreement both methods = 90.32%
		• axillary folds,					
		• pelvic girdle,	• Columnar Profile (PC)				
		• inter shoulder blade most prominent point of the spine (T5),					
		• less prominent lumbar spine point (L3),					
		• the start point of the gluteal fold,					
		• point in the neck base level (C7),					
		• most prominent points of the shoulder blades,					
		• least prominent points on lumbar pit					
Pino-Almero et al., 2017a [29]	88 children with juvenile or adolescent idiopathic scoliosis, 12 boys and 76 girls, 7–17 years old	• shoulder corners,	• Posterior Trunk Symmetry Index (POTSI),	• comparison topographic and radiographic variables (asymmetry of the back and Cobb angle, respectively)	• Pearson’s linear correlation coefficient (r)		• revealed significant correlations (p ≤ 0.01) between the Cobb angle with DHOPI (r = 0.810) and POTSI (r = 0.629)
		• axillary folds,	• Horizontal Plane Deformity Index (DHOPI),				
		• pelvic girdle,					
		• most prominent point in the central axis of thoracic spine (T5 vertebrae),	• Columnar Profile (PC)				
		• less prominent point in the central axis of lumbar spine (L3)					
		• home natal cleft (sacral),					
		• base of the neck (C7),					
		• most prominent point in shoulder blades,					
		• most prominent point in the lumbar graves,					
		• most prominent point in the buttocks					
Pino-Almero et al., 2017b [30]	155 patients, 7−21 years old divided into two group:	• shoulder corners,	• Posterior Trunk		• False Positive Rate (FPR, %),	• Intraobserver and interobserver reproducibility–Interclass Correlation Index	• Interclass Correlation Index (DHOPI = 0.983, POTSI = 0.959, PC = 0.984; intraobserver measures),
	• 88 patients with scoliosis,	• axillary folds,	• Symmetry Index (POTSI),		• False Negative Rate (FNR, %),		• Interclass Correlation Index (DHOPI = 0.987, POTSI = 0.978, PC = 0.969; interobserver measures),
	• 67 patients (control group)	• pelvic girdle,	• Horizontal Plane Deformity Index (DHOPI),		• Positive Predictive Value (PPV, %),		• FPR = 26%,
		• most prominent point in the central axis of thoracic spine (T5 vertebrae),	• Columnar Profile (PC)		• Negative Predictive Value (NPV, %),		• NR = 7.96%,
		• less prominent point in the central axis of lumbar spine (L3),			• the sensitivity,		• PPV = 86.17%,
		• home natal cleft (sacral),			• the specificity		• NPV = 84.08%,
		• base of the neck (C7),					• sensitivity = 92.04%,
		• most prominent point in shoulder blades,					• specificity = 74%
		• most prominent point in the lumbar graves,					
		• most prominent point in the buttocks					
Kuroki et al., 2018 [31]	689 293 children, boys and girls, 7−12 and 13−15 years old		• Moiré fringes on each half of the back,	• inspection by school doctors or nurses for students and radiograph	• Positive Predictive Value (PPV, %)		• PPV = 7.6% in 8th grade,
			• shoulder line,				• PPV = 2.1% in 5th grade (scoliosis greater than 20°)
			• waist line,				
			• scapular height				

MT- Moiré topography.

Seven studies compared MT method to radiographic Cobb angle [24, 25, 27–31] and one study compared MT method to scoliometer [26]. Studies investigated validity by Spearmen correlation [24], or The False-Positive Rate [25], Positive Predictive Value [26, 27, 30, 31], Negative Predictive Value [26, 30], the sensitivity and the specificity [26, 30] and by Pearson’s linear correlation coefficient [29, 30]. Three studies investigated both validity and reliability [26, 29, 30]. The repeatability of the MT examination was assessed based on the value of intraobserver and interobserver error [26] and Interclass Correlation Index [28, 30].

### Methodological quality assessment

The average quality of the 8 studies was 67% (range 62%-78%). All disputes were solution during a consensus meeting. Table 2 shows the results of the methodological quality assessment. All studies were of high quality, scoring ≥ 60% on the critical appraisal tool. The main items with low scores were an evaluator’s characterization (75% of studies unreported), a period of time between the test collection (75%), and a description of cases of sample loss (50%).

### Anatomical markers and evaluation parameters

The reference markers most commonly used were: the spinous processes from C7 to S1 [24, 26, 28–30], lower angles of the scapulas [24], corners of the shoulders—right and left [28–30], axillary folds—right and left [28–30], pelvic girdle—right and left [28–30], inter shoulder blade most prominent point of the spine (T5) [28–30], less prominent lumbar spine point (L3) [28–30], the start point of the gluteal fold [28–30], most prominent points of the shoulder blades—right and left [28–30], least prominent points on lumbar pit—right and left [28–30], and posterior superior iliac spines (PSIS) [24, 26], as suggested by the Society on Scoliosis Orthopedic and Rehabilitation Treatment (SOSORT) [32]. Three studies did not use markers.

Posterior Trunk Symmetry Index (POTSI), Horizontal Plane Deformity Index (DHOPI), and Columnar Profile (PC) were the most commonly used parameters [28–30]. Also, the following parameters were analyzed: maximum deflection of spinous process line from the line C7-S1 (UK) [24], asymmetry of the waistline and deviation of the dorsal side of the thoracic spine (α) [25], upper thoracic spine [25], thoracic spine [25], lumbar spine [25], shoulder height [27], pelvic tilt [27], waistline differences [25, 27], Surface Trunk Rotation (STR) [26], Moiré fringes on each half of the back [26, 27].

### Validity and reliability

#### Reliability and comparison of radiographic and scoliometer with MT measurements

The repeatability of the MT examination was assessed based on the value of the intraobserver and interobserver error [26] and the Interclass Correlation Index [28, 30]. MT evaluation has good repeatability (intraobserver error = 1.9°, interobserver error = 0.8° for the Surface Trunk Rotation (STR) parameter [26]. Two studies reported very high intraobserver and interobserver correlation (the Interclass Correlation Index: DHOPI = 0.983, POTSI = 0.959, PC = 0.984 for intraobserver measures; DHOPI = 0.987, POTSI = 0.978, PC = 0.969 for interobserver measures) [28, 30]. Table 3 shows study characteristics.

#### Validity

Correlation between MT parameters and radiographic Cobb angle ranged from moderate to high (Table 3). The values of Spearman correlation indicated high or moderate correlations (0.61–0.78) taking into account particular spinal segments [24]. High correlations were observed in thoracic and lumbar scoliosis, and moderate correlation in thoracolumbar scoliosis [24]. Two studies reported high or moderate and significant correlations (p ≤ 0.01 between the Cobb angle with DHOPI and POTSI) [28, 29].

The validity of the MT examination assessed by False Positive Rate (FPR) reported the screening test’s false-positive rate ranged from 32.4% [25] to 66.7% [27] participants. The validity of the MT examination assessed by Positive Predictive Value (PPV) or Negative Predictive Value (NPV) reported PPV ranged from 33.3% [27] to 86.17 [30]. One study examined by MT the predictive value of school scoliosis screening (SSS) in order to detection a curve of over 20° [31]. The value was respectively 2.1% for fifth-grade students and 7.6% for eighth-grade students.

In two studies one of the objectives was to evaluate the accuracy of MT as a screening tool by determining sensitivity and specificity. One study compared the MT method to scoliometer [26] and reported that STR value provided an unsatisfactory sensitivity and specificity (STR ≥ 5: the sensitivity = 64.5%, the specificity = 88%; STR ≥ 4: the sensitivity = 77.4%, the specificity = 71.1%) [26]. Another study reported a perfect sensitivity (92.04%) and allowed specificity (74%) [30].

## Discussion

The aim of this systematic review was to analyze the current knowledge regarding the reliability and validity of Moiré topography as a screening and diagnostic tool. It is known and used mainly in Japan (the most advanced country in using this method) [25, 27, 31], Poland [24, 26] and Spain [28–30]. This systematic review shows that MT has many advantages. Studies showed that MT had high repeatability and high intraobserver and interobserver correlation, and correlation between MT parameters and radiographic Cobb angle ranged from moderate to high.

On the other hand, studies showed the following disadvantages of the MT method in scoliosis screening (Table 4). One study reported hardship in determining the cut-off values for the STR parameter and low sensitivity and specificity of MT method. Moreover, the studies did not reveal the advantage of MT as a screening method in the detection of idiopathic scoliosis in comparison to radiograph [30] or clinical examination with the use of the scoliometer [26].

**Table 4:** Main conclusions from studies included in the systematic review.

Study	Exposure	Outcome	Main conclusions
Fugiel and Krynicka, 2010 [24]	MT and radiograph	Validity	MT method may be used only for the purpose of screening studies carried out in order to diagnose postural defects.
Ueno et al., [25]	MT and radiograph	Validity	The existence of a large number of false positives results is a very serious problem that occurs in most of the described screening programs in schools, as a result of the referral of a relatively large number of children. Primary screening for MT is beneficial because it does not require doctors, and the time that children have to spend in the actual screening process is negligible.
Chowanska et al., 2012 [26]	MT and scoliometer	Validity and reliability	Studies have not shown an advantage of surface topography as a screening method to detect idiopathic scoliosis compared to a clinical trial using scoliometer. The lack of STR value provided a satisfactory sensitivity and specificity at the same time.
Yamamoto et al., 2015 [27]	MT and radiograph	Validity	The MT school screening test had a high false-positive rate. The study highlighted the need for further research into reducing the false-positive rate of MT in scoliosis screening.
Pino-Almero et al., 2016 [28]	MT and radiograph	Validity and reliability	A significant correlation was found between the changes in DHOPI, POTSI, and the Cobb angle. By obtaining the correlation of variables connected DHOPI and POTSI and the Cobb angle, can be monitored the progression of scoliosis. This would help to reduce the number of exposures to ionizing radiation.
Pino-Almero et al., 2017a [29]	MT and radiograph	Validity	Although the MT method cannot replace radiographs in the diagnosis of scoliosis, the correlations between radiographic and topographic parameters suggest that it offers additional quantitative data that can complement the radiological examination.
Pino-Almero et al., 2017b [30]	MT and radiograph	Validity and reliability	The MT method may be less sensitive in low-grade scoliosis with a slight rotational component but of little importance.
			The MT method may be a useful test in the screening phase of idiopathic scoliosis with a higher sensitivity than the Adams test and similar specificity.
Kuroki et al., 2018 [31]	MT and radiograph	Validity	School screening for scoliosis with MT appeared to be effective in detecting scoliosis, although both the positive predictive value and the benchmark for second screening were low.

MT–Moiré topography; STR–Surface Trunk Rotation; POTSI—Posterior Trunk Symmetry Index; DHOPI—Horizontal Plane Deformity Index.

A very serious problem is the occurrence of a large number of false positives results. The authors suggested that the high rate of false-positive results was probably due to the fact that the control samples included patients referred for possible scoliosis, and most of them had some kind of asymmetry in their backs, which was detected by the MT method [30]. Other studies suggested that the problem of false-positive results could be minimalized by rescreening [25] and emphasized the need for further research on reducing the false-positive rate of MT in scoliosis screening [27].

Although MT cannot replace radiography in the diagnosis of scoliosis, the authors concluded that the MT method offers additional quantitative data that can complement the radiological examination [28–30]. Studies suggested that a combined examination (e.g., MT combined with Cobb angle) improved the accuracy of screening results. Thanks to the correlation of the combined DHOPI and POTSI variables with the Cobb angle, it is possible to monitor the progression of scoliosis, and to reduce the number of exposures to ionizing radiation, and is a harmless procedure that can be applied repeatedly.

In order to eliminate the risk of measurement error, the same methodological procedure should be developed. Many studies suggested that body posture should be assessed at the same time of day [24, 33] by a qualified person (physiotherapist, doctor, radiologist, teacher) with several years of experience [24, 34–36]. The room in which the examination is performed should be darkened [35–39]. Only one article drew attention to the fact that the height of the measuring station should be adjusted to the child’s height [26]. Several studies indicate that the selection of the best view of the child’s body is used to analyze deviations in body posture [24, 38, 40, 41]. In two studies, the authors reported that a dermograph was used to mark bone points on a child’s body [42, 43].

However, the assessment of the generated topograms on the basis of visual inspection and marking specific points on the patient’s bone body, may affect the accuracy of the measurements [7, 19, 44]. Due to the fact that the topogram is made in strictly defined conditions (i.e., constant distance, the exact position of the level, constants lighting conditions, the same parameters of the optical system) [8], this measurement should be repeatable.

When analyzing the accuracy of the measurement, attention should be paid to the specificity of the object being tested, which is the human body. Practically, it is not possible to show physiological points on the patient’s skin with an accuracy greater than 5 mm. Taking this into account, the parameter, e.g., the difference in the height of the blade angles, is affected by a random error of 1 cm. For this reason, it should be assumed that the value of 1 cm is the limit of the accuracy of the method due to physiological features. On the other hand, the accuracy of the apparatus is 1 mm (resolution and the value of the parameters calculated), so it is 10 times better than that necessary from the point of view of physiology. This is of practical importance, as it allows one to detect the first features of curvatures still invisible to the naked eye [19, 44, 45]. MT has been shown to be more effective than simply reviewing posture for verification symmetries of the cervical and lumbar spine [46]. Moreover, the studies showed the evolution of the MT method, reflecting the increasing efforts to improve the accuracy and precision of the method [19, 44].

Despite the advantages of using this technique, there is a high probability of errors in evaluating a large number of topograms qualitatively. The evaluation of the topograms produced is based on visual inspection of the images. It can be a difficult task. Fatigue caused by conducting many subjective evaluations can interfere with the assessment of the images. Moreover, the difficulty may lie in the different ways of assessing posture, the method of recording the results, using measuring tools with a different structure, and the definition of the parameters measured. Standardization should therefore include the necessary measurement conditions, the unification of measurement tools, and typing the definition of the measured attitude traits to overcome terminological barriers.

A literature review showed that the consistency assessment regarding the asymmetry in the sagittal plane was slightly smaller than in the other planes. These less reliable measurements of the spine in the sagittal plane can be justified, as most indirect methods are concerned with the univariate assessment and spine lesions are usually three-dimensional [45, 47]. Although the MT does perform a three-dimensional analysis, it may be difficult for the assessor to see asymmetries in the sagittal plane due to the smaller sharpness of the fringes, especially in the cervical and lumbar regions [7].

Scoliosis screening is the most important factor preventing deformity progression. MT has been applied in school screening programs [25, 27, 28]. Scoliosis is studied by looking at asymmetric patterns in the Moiré photo. However, it only provides qualitative results, that is, whether the patient has scoliosis or not, and the exact Cobb angle cannot be inferred from the fringe patterns. It was not possible to estimate the value of the Cobb angle despite the high accuracy (AUC = 0.929) of the application of the diagnostic system based on artificial intelligence to screening scoliosis using the Moiré image [44].

Studies suggested that MT was confirmed to be a method for the easy detection of low angular value scoliosis (Cobb angle < 10°) [8] and more accurate results are obtained with small spine deformities than large spine deformities [44]. Most studies of the prevalence of scoliosis detected scoliosis with a higher angular value (higher than 10°), which limits early intervention by healthcare professionals, because it determines the scoliotic process is the vertebral rotation [8]. The authors claimed that MT is the most sensitive to this factor. However, the Scoliosis Research Society (SRS) suggests confirming the diagnosis at a Cobb angle of 10˚ [32]. Therefore, it can be said that the authors’ methodological assumption was incorrect.

This systematic review showed that there is moderate evidence for the reliability and validity of MT as a screening and diagnostic tool. Nowadays alternatives to MT are methods as raster stereography (the Formetric 4D) [14], 3D ultrasound imaging (the Scolioscan system) [16], and Infrared Thermography (IR thermography) [17]. Studies showed that these methods had a good reproducibility [14, 15, 17] and had good to excellent correlation comparison with radiography [14, 15]. However, the authors suggest that further studies are required to demonstrate their clinical values with a larger number of scoliosis patients with different types of curvature [14, 15].

### Strengths and limitations

This is the first review focused only on one non-radiographic method of measuring body posture. This systematic review looked at the sensitivity of only high quality research However, there are some limitations to this review. Studies with significant heterogeneity were summarized. We found heterogeneity between studies with regard to aspects such as study population, exposure assessment methods, and data presentation that may limit the final conclusions. A quantitative meta-analysis could not be performed. Finally, the search strategy was limited only to full-text articles in English.

## Conclusion

Based on the evidence from eight studies, there is moderate evidence for the reliability and validity of Moiré topography as a screening and diagnostic tool. MT is an alternative method of examining the deformity of the spine and trunk but there is still no strong evidence for the accuracy of MT. Moreover, the methodology of MT should be standardized in order to use it as an accurate screening tool. Therefore, researchers should be careful in drawing conclusions from studies using the MT measurement.

## Supporting information

S1 Checklist PRISMA 2020 checklist. (DOCX)
